# Phenolic Compounds and Antioxidant Properties of Fermented Beetroot Juices Enriched with Different Additives

**DOI:** 10.3390/foods13010102

**Published:** 2023-12-28

**Authors:** Karolina Jakubczyk, Klaudia Melkis, Katarzyna Janda-Milczarek, Karolina Skonieczna-Żydecka

**Affiliations:** 1 Department of Human Nutrition and Metabolomics, Pomeranian Medical University in Szczecin, 24 Broniewskiego Street, 71-460 Szczecin, Polandkatarzyna.janda.milczarek@pum.edu.pl (K.J.-M.); 2 Department of Biochemical Science, Pomeranian Medical University in Szczecin, 71-460 Szczecin, Poland; karolina.skonieczna.zydecka@pum.edu.pl

**Keywords:** beetroot, fermented beetroot juice, fermentation, antioxidant, antioxidant properties, spices

## Abstract

Fermented beetroot juice is a beverage obtained from the fermentation of beetroot, most commonly red beet (*Beta vulgaris* L. *var. conditiva*). Nowadays, this product is increasingly recognised as a functional food with potentially beneficial health properties. It has been suggested to have antioxidant, anti-inflammatory, anticancer, antihypertensive, immunomodulatory, and probiotic effects, among others. Moreover, with the increasing popularity of the drink, newer variants are appearing in the food market, obtained by modifying the traditional recipe, adding other raw materials, herbs, and spices. Therefore, the aim of this study was to evaluate and compare the antioxidant potential and phytochemical composition of the selected fermented beetroot juices in different flavour variants available in the Polish food market. The study material consisted of six fermented beetroot juices: traditional, with garlic, with horseradish, with acerola, without salt, and iodized. The obtained results showed that the addition of acerola, horseradish, garlic, salt, and iodine in the form of sodium iodide and potassium iodide influenced the composition and properties of fermented beetroot juice. The most promising product in terms of potentially beneficial health properties related to the prevention of free radical diseases was fermented beetroot juice without salt (FRAP—5663.40 µM Fe (II)/L, ABTS—96.613%, TPC—760.020 mg GAE/L, TFC—221.280 mg RE/L). Iodized fermented beetroot juice had the highest vitamin C content—51.859 mg/100 mL. However, all the products tested were characterised by a significant content of biologically active substances with antioxidant properties and showed a high antioxidant potential. Moreover, all the fermented beetroot juices were rated positively in terms of flavour intensity, sweetness, acidity, colour, and overall acceptability. They can, therefore, be a good source of antioxidants in the daily diet.

## 1. Introduction

Fermented vegetable juice is a liquid obtained by the fermentation of selected raw vegetable materials. The process is based on fermentation under anaerobic conditions, where appropriate bacteria from the *Lactobacteriaceae* family convert simple sugars into lactic acid [1]. This method has been used for centuries in food processing to extend the shelf life of, for example, fruit or vegetables, to preserve food, and to improve its organoleptic properties.

In many households, fermented vegetable beverages are made by natural/spontaneous fermentation or by adding a slice of rye bread as a source of lactic acid bacteria [2]. In addition, to ensure the safety and stability of the process, the raw material is cut into smaller pieces and placed in a glass jar that has been previously boiled, over which a salt solution, known as brine, is poured [1]. Unfortunately, these methods do not allow for production to be standardised on a large scale. Therefore, the use of commercial starter cultures is becoming more widespread in order to obtain final products with predictable and reproducible properties and sensory characteristics [2,3,4]. In contrast to spontaneous fermentation, the use of inoculum produces a beverage with a higher content of probiotics, microorganisms, and a lower pH, which reduces the likelihood of the development of pathogenic microorganisms [1,2,3,5,6].

One well-known fermented vegetable beverage is based on beetroot (*Beta vulgaris* L. *var. conditiva*). It is known as beet kvass, beet sourdough, fermented beet juice, pickled borscht, and pickled beet juice, depending on the country and translation. It is characterised by a reddish-purple colour and a refreshing, distinctive sweet–sour taste and smell [7]. It is particularly popular in Central and Eastern Europe, where it is the main ingredient in traditional borscht, or beetroot soup. According to the traditional recipe, the marinade is enriched with allspice, bay leaves, and garlic to improve the flavour. Today, fermented beetroot juice is gaining popularity as a functional food.

Scientific evidence suggests that fermented beetroot juice has potent antioxidant, anti-inflammatory, anticancer, antimicrobial, antidepressant, antihypertensive, hepatoprotective, hypocholesterolemic, immunomodulatory, and probiotic properties [3,8,9,10,11,12,13,14].

The range of beneficial biological effects that fermented beetroot juice can have on consumers is directly related to the valuable composition of *Beta vulgaris* L. and the metabolites produced during the fermentation process. Beetroot and its products are a valuable source of nutrients, such as proteins, carbohydrates, dietary fibres (pectins, oligosaccharides), minerals (iron, magnesium, calcium, potassium, zinc, sodium, phosphorus, copper), vitamin C and B group vitamins (folic acid, B_7_ or B_3_), as well as numerous amino acids, nitrates, and biogenic amines [6,8,9,15]. They are also known for their significant content of phytochemicals with strong antioxidant properties, including polyphenols and pigment compounds such as carotenoids and betalains [16,17]. It is, therefore, believed that the consumption of beetroot and beetroot products can prevent the adverse effects of oxidative stress, thereby protecting against the occurrence of chronic diseases and free radical-mediated diseases [18].

However, it should be noted that the content and quantity of the individual biochemical components are not fixed. The chemical and microbiological profile of fermented beetroot juice is influenced by the variety and maturity of the beet, the fermentation method and parameters (time, temperature), the composition of the starter cultures, the addition of other raw materials or spices, and the duration and conditions of storage [1,6,19,20].

In order to obtain a product with the most favourable nutritional and sensory characteristics, food manufacturers are offering consumers ever newer product proposals, obtained by modifying traditional recipes and adding other raw materials, herbs, and spices. Unfortunately, there is currently a lack of studies dedicated to the analysis of the properties of unconventional juices from fermented beetroot, which indicates the need for further research.

Therefore, the aim of this study was to evaluate, for the first time, the antioxidant potential and phytochemical composition of the selected fermented beetroot juices in different flavour variants available in the Polish food market, and to compare and identify the best product in terms of the potential health benefits related to the prevention of the adverse effects of oxidative stress.

## 2. Materials and Methods

### 2.1. Materials

**Figure 1:**
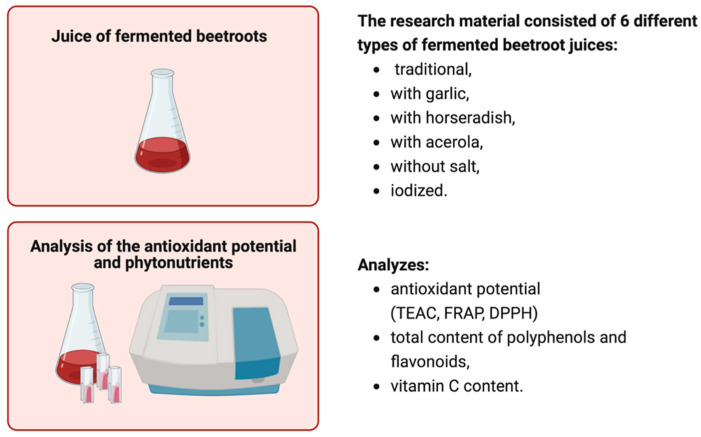
Materials and methods.

The research materials consisted of six variants of fermented beetroot juice (*Beta vulgaris* L.): traditional, with added garlic, with added horseradish, with acerola, without salt, and iodized (Figure 1). The flavour additives were added before the fermentation process of the beetroots. All the products were obtained from an organic producer of preserves (Delikatna.bio, Gdańsk, Poland). The composition and nutritional values derived from the labels of the products tested are presented in Table 1.

### 2.2. The Determination of the Total Polyphenol Content (TPC)

Total polyphenol content (TPC) was determined according to, the method of Singleton V.L., Rossi J.A. using the Folin–Ciocalteu reagent (Chempur, Poland) [21]. The absorbance was measured at 765 nm (Agilent 8453UV, Santa Clara, CA, USA). All the assays were performed in triplicate. The content of polyphenols was determined from the calibration curve using gallic acid (GAE) as the reference standard (0, 10, 20, 30, 40, 50, 75, 100, and 200 mg/L of gallic acid).

### 2.3. The Determination of the Total Flavonoid Content (TFC)

The total flavonoid content was determined according to the methods of Hu et al. [22]. Different concentrations of rutin were used for plotting the standard calibration curve. The content of flavonoids was determined from the calibration curve using rutin equivalent to the reference standard (0–120 mg/L of rutin equivalent). The absorbance was measured at 510 nm (Agilent 8453UV, Santa Clara, CA, USA). All the assays were carried out in triplicate.

### 2.4. The Determination of the Ferric Ion Reducing Antioxidant Power (FRAP) Method

The FRAP method, used to determine the total reduction potential, is based on the ability of the sample to reduce Fe^3+^ ions to Fe^2+^ ions. The FRAP instrument determined the ability of the sample to reduce 1 mol of Fe^3+^ to Fe ^2+^ [23,24]. The absorbance was measured at 593 nm (Agilent 8453UV, Santa Clara, CA, USA). All the assays were performed in triplicate. The ferric reducing antioxidant power was determined from the calibration curve using Fe(II)/L as the reference standard (0, 50, 100, 200, 300, 400, 500, 600, 700, 800, 900, 1000, 2000, 3000, 4000, and 5000 µM Fe(II)/L).

### 2.5. Antioxidant Activity using the DPPH Method

The antioxidant activity of the samples was measured by the spectrophotometric method using the synthetic radical DPPH (2,2-diphenyl-1-picrylhydrazyl, Sigma, Poznań, Poland), according to Brand-Williams et al. and Pekkarinen et al. [25,26]. The spectral absorbance was measured immediately at 518 nm (8453UV, Agilent Technologies, Santa Clara, CA, USA). All the assays were performed in triplicate. The results are expressed as the percentage of the DPPH radical inhibition.

The antioxidant potential (antioxidant activity, inhibition) of the tested solutions was expressed by the percentage of DPPH inhibition using the following formula.

$$\%\;inhibition=\frac{A0-As}{A0}\;\times \;100$$

where

*A*_0_—the absorbance of the DPPH solution at 518 nm without the tested sample.

*A*_s_—the absorbance of the DPPH solution at 518 nm with the tested sample.

### 2.6. Antioxidant Activity using the ABTS Method

The antioxidant activity of the samples was determined by the spectrophotometric method using the ABTS reagent (2,2’-azobis(3-ethylbenzothiazolin-6-sulfonate, Sigma, Poznań, Poland). The spectral absorbance was measured immediately at 750 nm (8453UV, Agilent Technologies, Santa Clara, CA, USA). All the assays were performed in triplicate. The results are expressed as the percentage of the inhibition of the DPPH radicals.

The antioxidant potential (antioxidant activity, inhibition) of the tested solutions was expressed as the percentage of ABTS inhibition using the following formula.

$$\%\;inhibition=\frac{A0-As}{A0}\;\times \;100$$

where

A_0_—the absorbance of the ABTS solution at 750 nm without the tested sample.

A_s_—the absorbance of the ABTS solution at 750 nm with the tested sample.

### 2.7. Determination of the pH

The pH of the fermented beetroot juices was determined using a pH metre (SCHOTT Instruments; SI Analytics Mainz, Mainz, Germany).

### 2.8. Determination of the Vitamin C Content

The determination of the vitamin C content was carried out according to ISO 6557-2:1984 [27]. In this method, 2,6-dichlorophenoloindophenol (2,6-DCPIP, Sigma, Poznań, Poland) was added to a sample, reacted with vitamin C, and after extraction with xylene, its excess was determined spectrophotometrically. The absorbance measurements were taken at 500 nm in 1-cm quartz cuvettes with xylene as a reference. The measurements were taken on the Agilent 8453 UV–VIS spectrophotometer (8453UV, Agilent Technologies, Santa Clara, CA, USA). In this work, the following chemicals were used: o-xylene of spectrophotometric grade, 98% (SIGMA, Sigma, Poznań, Poland, 2,6-dichlorophenolindophenol sodium salt hydrate (SIGMA, Sigma, Poznań, Poland), and the remaining chemicals were of an analytical grade. The concentration of vitamin C was expressed in mg of vitamin C per 100 mL of fermented beetroot juice (mg/100 mL).

### 2.9. Sensory Evaluation

Sensory evaluation tests were performed by a panel of 20 trained members. The expert team consisted of 8 men and 12 women; all of them had experience with fermented beetroot juice. Before assessing the fermented beetroot juices, the panelists were trained to recognise all the sensory descriptors (aroma, sweetness, acidity, colour, overall acceptability). The descriptors were developed for this experiment. They were developed on the available scientific publications for fermented beetroot juice and other fermented beverages where they are commonly used. The panellists’ training was designed to confirm the sensitivity, ability, and consistency of the panellists in assessing the appearance, mouthfeel, taste, and aroma of the sample. The assessment was carried out individually under white lights and in an air-conditioned room. In all cases, the samples were presented randomly and were served at an ambient temperature in coded clear plastic glasses. Potable water was available for rinsing the mouth between the test samples. The test products were evaluated using a scoring method in terms of perception of the aroma intensity, sweetness, acidity, colour, and overall acceptability. A scoring range of 1–9 was used, where 1 indicated extreme dislike, 2—great dislike, 3—moderate dislike, 4—slight dislike, 5—neither liking nor dislike, 6—slight liking, 7—moderate liking, 8—great liking, 9—extreme liking.

### 2.10. Statistical Analysis

A statistical analysis was performed using the MedCalc^®^ statistical software version 20.218 (MedCalc Software Ltd., Ostend, Belgium; https://www.medcalc.org; 2023) and Microsoft Excel 2017. The results were expressed as the median, upper, and lower quartiles, and minimum and maximum values. The non-parametric Kruskal–Wallis test with Conover’s post-hoc test was used to assess the differences between the study parameters. Spearman’s test was used to calculate the correlation coefficient. Differences were considered significant when *p* ≤ 0.05.

## 3. Results

### 3.1. Analysis of the Antioxidant Properties of Fermented Beetroot Juices

The antioxidant activity of the selected fermented beetroot juices was evaluated on the basis of their ability to reduce iron ions (FRAP), their ability to neutralise radicals (DPPH, ABTS) and their total polyphenol (TPC), flavonoid (TFC), and vitamin C contents.

The antioxidant potential values of the tested products, expressed as their ability to reduce iron ions, ranged from 5253.40 to 5761.80 µM Fe (II)/L. The highest value was recorded for the fermented beetroot juice without added salt, and the lowest for the traditional fermented beetroot juice. Statistically significant differences between the tested juices were evaluated in post-hoc analyses, as depicted in Table 2.

The antioxidant potential values of the fermented beetroot juices tested, expressed as a percentage of the ABTS radical inhibition, ranged from 37.936 to 96.708%. The highest value was recorded for fermented beetroot juice without added salt, and the lowest value was that of fermented beetroot juice with horseradish. Statistically significant differences were observed between all the fermented beetroot juices (Table 3).

The antioxidant potential values of the tested products, expressed as a percentage of the DPPH radical inhibition, ranged from 54.756 to 79.066%. The traditional fermented beetroot juice had the highest recorded value, while the fermented beetroot juice without added salt had the lowest. Statistically significant differences were observed between all the starters except for traditional fermented beetroot juice vs. fermented beetroot juice with garlic and fermented beetroot juice with horseradish vs. fermented beetroot juice with garlic (Table 4).

The total polyphenol content (TPC) of the fermented beetroot juices tested varied between 107.77 and 790.45 mg GAE/L. The fermented beetroot juice without added salt had the highest values for this parameter, while the fermented beetroot juice with garlic had the lowest values. Statistically significant differences were observed between all the fermented beetroot juices except for traditional fermented beetroot juice vs. fermented beetroot juice with horseradish and traditional fermented beetroot juice vs. fermented beetroot juice with garlic (Table 5).

The total flavonoid content (TFC) of the fermented beetroot juices tested ranged from 83.922 to 223.420 mg RE/L. The highest total flavonoid content was again found in fermented beetroot juice without added salt and the lowest in the iodized fermented beetroot juice. Statistically significant differences were observed between all the fermented beetroot juices except iodized fermented beetroot juice vs. fermented beetroot juice with garlic, traditional fermented beetroot juice vs. fermented beetroot juice with acerola, and traditional fermented beetroot juice vs. fermented beetroot juice with horseradish (Table 6).

Fermented beetroot juice also appeared to be a good source of vitamin C. The highest concentration was found in iodized fermented beetroot juice at 51.859 mg/100 mL, while the lowest concentration was found in fermented beetroot juice without added salt at 29.927 mg/100 mL. No statistically significant differences were observed for iodized fermented beetroot juice vs. fermented beetroot juice with horseradish, fermented beetroot juice with acerola vs. fermented beetroot juice with horseradish, fermented beetroot juice with acerola vs. fermented beetroot juice with garlic, and fermented beetroot juice with horseradish vs. fermented beetroot juice with garlic (Table 7).

**Figure 2:**
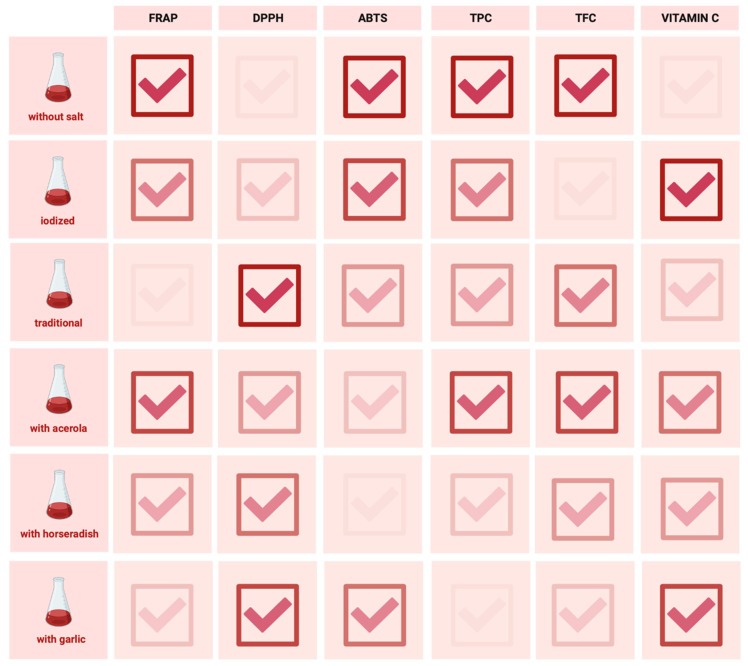
The comparison of the antioxidant properties of the tested juices of fermented beetroots. The value of the tested parameters was determined by changing the color intensity.

The fermented beetroot juice without added salt had the highest antioxidant potential, as shown in Figure 2. The antioxidant content of this product, both polyphenols and flavonoids, was higher than that of the other products. It also had the highest antioxidant potential tested using ABTS and FRAP. Iodized fermented beetroot juice proved to be the best source of vitamin C.

### 3.2. The Analysis of the pH

The pH values of the tested beverages were not statistically significantly different between each other. All the beetroot juices had pH values close to each other, ranging from 3 to 4 (Table 8).

**Figure 3:**
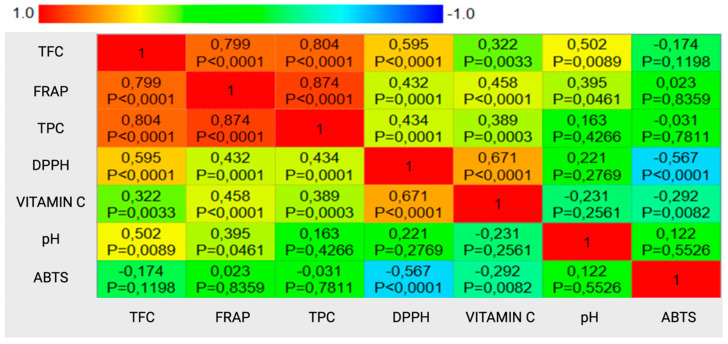
Spearman’s rank correlations between the tested parameters for the different types of fermented beetroot juice. Created with MedCalc^®^ Statistical Software version 20.218.

The statistical analysis of the results showed statistically significant (for *p* ≤ 0.05) correlations between the fermented beetroot juices parameters studied, as shown in Figure 3. A very strong positive correlation was observed between the concentration of polyphenols and the antioxidant potential measured using the FRAP method. Strong positive correlations were also observed between the total flavonoid content and the number of polyphenols, as well as the antioxidant potential measured using the ferric ion reducing ability (FRAP). A negative correlation was found between DPPH and ABTS.

### 3.3. Sensory Evaluation

**Figure 4:**
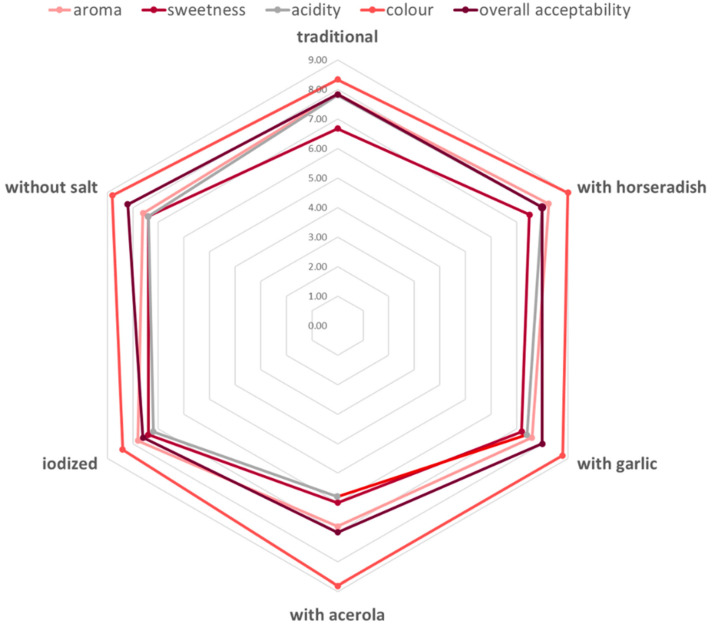
Sensory evaluation of the fermented beetroot juices.

A sensory evaluation was also conducted on a 10-point scale, where 10 represented the most desirable characteristic and 1 represented the least desirable. The flavour, sweetness, acidity, colour, and overall acceptability were taken into consideration. The highest score in terms of aroma, sweetness, acidity, and colour was achieved by fermented beetroot juice with horseradish, but fermented beetroot juice without salt turned out to be the best in terms of overall acceptability. However, differences between tested fermented juices were not statistically significantly. All the fermented beetroot juices were evaluated very positively (Table 9, Figure 4).

## 4. Discussion

The fermented beetroot beverage is a fermented product that is becoming increasingly popular with consumers. It owes its popularity to its rich content of phytochemicals, which have many health benefits, including the ability to scavenge free radicals.

One of the biggest problems of the modern world, responsible for several diseases, is an unhealthy diet [28]. The results of numerous scientific studies suggest that foods rich in antioxidants correlate with a lower incidence of free radical-mediated diseases and associated mortality [29,30,31].

Beetroot (*Beta vulgaris* L.) is considered one of the best plant sources for antioxidants. Although this vegetable is rich in many bioactive compounds, the main group of compounds that determine its ability to scavenge free radicals are betalains [17]. Betalains are natural, water-soluble pigment compounds found in only ten families of the carnation order (*Caryophyllales*), including the Grevilleas, to which beetroot belongs. They owe their colour to a peculiar chromophoric arrangement of three double conjugated bonds. The group consists of red-violet betacyanins and yellow-orange betaxanthins. The most common betacyanin is betanin [32,33].

During beet fermentation, the compounds present in the raw material are released into a liquid as a result of the action of several factors, such as the metabolic activity of the microorganisms or the composition and osmolality of the marinade. In addition, the fermentation process increases the bioavailability of the phytonutrients in the final product due to the metabolic activity of the various microorganisms present in the solution [34,35,36]. This is due to the enzymes released by microorganisms, including cellulolytic, ligninolytic, and pectinolytic enzymes, which are capable of hydrolysing the ester and glycosidic bonds of the cell walls of plant tissues, thereby degrading them and releasing the bioactive compounds bound to the insoluble matrix [5,37,38]. Bacterial enzymes are also responsible for the partial degradation of complex molecules, e.g., polyphenols into simpler compounds with greater biological activity [39,40]. Therefore, the fermentation process may lead to an increase in the total content of compounds with antioxidant properties [41]. Panghal et al. compared fresh beetroot juice with lacto-fermented beetroot juice. They found that the fermentation process resulted in an increase in polyphenols, flavonoids, and antioxidant activity in beetroot juice [42].

All this shows that traditional fermented beetroot juice has excellent antioxidant properties. They owe this in particular to the betacyanins (betanin, betanidin, neobetanin, isobetanin, isobetanidin), whose abilities are probably determined by the free phenolic hydroxyl groups in their structure. The antioxidant potential of the products concerned is also influenced by polyphenolic compounds, including phenolic acids (e.g., protocatechuic acid, transcinnamic acid, isoferulic acid, p-hydroxybenzoic acid, m-hydroxybenzoic acid, syringic acid, and synapinic acid) and flavonoids (e.g., myricetin, isobutanidin, quercetin, kemferol, rutin hydrate, naringenin, hesperidin, catechin, epicatechin, epigallocatechin, among others) [37,43].

To the best of our knowledge, the present study was the first to analyse and compare the antioxidant properties of fermented beetroot juices obtained by modifying a conventional recipe. It was shown that fermented beetroot juices with more favourable antioxidant properties than conventional fermented beetroot juice are available in the food market and that the addition of acerola, horseradish, garlic, salt, and iodine in the form of sodium iodide and potassium iodide influences the composition and antioxidant potential of the ferments.

The antioxidant activity was assessed by the ability to reduce iron ions (FRAP), neutralise radicals (DPPH, ABTS), and the total polyphenols (TPC), flavonoids (TFC) and vitamin C.

The antioxidant potential values, expressed as a percentage of the ABTS radical inhibition, ranged from 37.936 to 96.708%. The highest value recorded was for fermented beetroot juice without added salt, while the lowest value was for horseradish. The antioxidant potential of fermented beetroot juice, measured using the FRAP method, ranged from 5253.40 to 5761.80 µM Fe (II)/L. The fermented beetroot juice without added salt also had the highest recorded value, while the traditional fermented beetroot juice had the lowest. The DPPH method, with results ranging from 54.756% to 79.066%, showed a negative correlation with the ABTS method. It showed the highest value for the traditional fermented beetroot juice and the lowest value for the fermented beetroot juice without added salt. The observed difference in the results was a consequence of certain possibilities and limitations of the methods used. The ABTS and FRAP methods allowed for the determination of the antioxidant capacity of both hydrophilic and lipophilic compounds [44]. In contrast, DPPH was only soluble in organic solvents, and therefore did not allow for the activity of hydrophilic compounds to be determined [45]. For this reason, scientific publications dealing with the antioxidant properties of plant products used these three methods together to make the results as reliable as possible. The data obtained suggested that in fermented beetroot juice without the addition of salt, the ability to neutralise free radicals was mainly provided by hydrophilic betalains, whereas in traditional fermented beetroot juice and with the addition of spices, the ability to neutralise free radicals was provided by hydrophobic antioxidants, i.e., carotenoids or flavonoids. Current knowledge suggests that the addition of spices to fermented substrates influences the microbial environment of the process, which in turn has a significant impact on the chemical profile and antioxidant properties. Consequently, in addition to improved palatability, a product with higher levels of bioactive compounds, e.g., isoflavones, and a higher antioxidant activity was obtained compared to a product produced without the addition of spices [46,47]. However, further research is needed to confirm the mechanism of influence and the effects of adding spices and other raw materials to fermented beetroot juice.

The fermented beetroot juices tested were also high in total polyphenols (107.77 to 790.45 mg GAE/L) and flavonoids (83.922 to 223.420 mg RE/L). In both cases, the fermented beetroot juice without added salt showed the highest values. In the traditional recipe, fermented beetroot juice is prepared by placing the selected raw material in a brine whose main component is NaCl. According to the literature, the addition of NaCl during the vegetable fermentation process has several functions. It lowers the water activity, increases the ionic strength of the solution, reduces the solubility of oxygen in water, and induces an increase in lactic acid production [38,48,49]. As a result, by creating unfavourable conditions for the growth of pathogenic bacterial microflora, it prevents spoilage, thereby ensuring the microbiological safety of the final product [49,50,51]. However, it is possible that this environment is also unfavourable for the growth of bacteria that positively influence the polyphenolic content of the resulting beverage. Therefore, subjecting beetroot to fermentation without the addition of salt has tangible benefits in terms of improved antioxidant properties. In addition, low-sodium products are now more desirable in the food market because they do not contribute to the development and occurrence of cardiovascular diseases, i.e., hypertension [52].

In order to evaluate the influence of different variables on the final antioxidant properties of red-beet-based products of two varieties, Choińska et al. tested and compared starters obtained from the spontaneous fermentation of the raw material and fermentation with single-strain or multi-strain starter cultures. Before fermentation, the whole beets were washed, peeled, and cut into 2–3 mm thick slices. The slices were then placed in sealed glass jars, soaked in a marinade (100 mL) containing salt (1.5%), and some were additionally inoculated with a fresh starter culture containing 1 × 105 CFU mL^−1^. The results of this study showed that the content of phenolic compounds in the juices obtained during the 7-day fermentation ranged from 524.1 to 766.0 mg GAE-L^−1^, depending on the variables. The antioxidant activity measured by the DPPH method was 0.26–0.67 μmol Trolox-mL^−1^, depending on the type of raw material, the type of fermentation and the starter cultures used [6].

Fermented beetroot juice also appeared to be a good source of vitamin C. Ascorbic acid is a powerful antioxidant, neutralising free radicals and protecting the body from oxidative stress. It also supports wound healing, participates in collagen synthesis, aids in iron absorption, participates in the synthesis of neurotransmitters, improves brain function, prevents and shortens infections, and has a positive effect on the cardiovascular system, preventing ischaemic heart disease and other cardiovascular dysfunctions [53,54,55]. As the human body is unable to synthesise and store it, it must be provided in sufficient quantities in the diet. In the products tested, its content ranged from 51.859 mg/100 mL to 29.927 mg/100 mL. Given that the recommended daily allowance (RDA) for vitamin C is 75 mg for women and 90 mg for men, drinking 100 mL of iodized fermented beetroot juice can cover 69.15% of the requirement for women and 57.62% for men [56]. Other products containing similar amounts of vitamin C are kimchi–51 mg/100 g fresh weight and cranberry juice 25 mg/100 mL [57,58].

It is also important to note the low pH of the fermented product. In our study, all the fermented beetroot juices had a similar pH, ranging from 3 to 4. The differences between the pH of the products tested were not statistically significant, which may indicate that the addition of acerola, horseradish, garlic, salt, and iodine in the form of sodium iodide and potassium iodide did not affect this parameter of the products obtained. Moreover, the beverages came from a commercial source where, presumably, fermentation was stopped when the fermented beetroot juices reached the above pH, and this parameter was strictly controlled. From a technological point of view, this ensures microbiological safety, as few pathogenic microorganisms are able to colonise this niche. Existing studies have shown that at pH > 4, betanin, the main pigment in beetroot, was approx. 1.5–2.0 times more active than some anthocyanins, which are known for their strong antioxidant properties [59]. The acidic environment created during the fermentation process also had a positive effect on the stability of ascorbic acid [60,61]. Thus, fermented beetroot juice seems to have pH values that are optimal for the high activity of the bioactive compounds they contain. On the other hand, from a medical point of view, regular consumption of a low pH beverage can have negative effects on the digestive system and even cause metabolic acidosis [62,63]. It is, therefore, advisable to use moderation and common sense when consuming fermented beetroot juice, as well as other ferments.

It should be noted that only a few studies have been published on the antioxidant properties of fermented beetroot juices. The scientific literature is much more likely to contain work on beetroot juice or beetroot itself. Fresh juice is characterised by the typical taste and smell of the raw material, described as bland and ‘earthy’ due to its geosmin content [7]. However, in an acidic environment, this compound is dehydrated to odourless argosmin. The fermentation process, therefore, helps to improve the sensory qualities, making the product more palatable. There is currently a lack of studies in the databases on the effect of the addition of individual raw materials on the composition and properties of fermented beetroot juice. As mentioned above, this study is pioneering.

## 5. Conclusions

The results presented showed that the addition of acerola, horseradish, garlic, salt, and iodine in the form of sodium iodide and potassium iodide influenced the composition and properties of fermented beetroot juice. The most promising product in terms of potential health-promoting properties related to the prevention of free radical diseases was fermented beetroot juice without salt. Iodized fermented beetroot juice had the highest vitamin C content. Further research is necessary to determine the exact impact of flavor additives to fermented beetroot juices on the properties and composition of the products. However, all the products tested were characterised by a significant content of biologically active substances with antioxidant properties and showed a high antioxidant potential. Moreover, all the fermented beetroot juices were rated positively in terms of flavor intensity, sweetness, acidity, colour, and overall acceptability. They can, therefore, be a good source of antioxidants in the daily diet.

## Figures and Tables

**Table 1:** Composition and nutritional value in 100 mL of the tested products.

Type of Fermented Beetroot Juice	Ingredients	Energy [kcal]	Fat of Which Saturates [g]	Carbohydrates of Which Sugars [g]	Protein [g]	Salt [g]
Traditional	Water, red beetroot*, garlic*, bay leaf*, allspice*, kłodawska salt	12	<0.1 (<0.1)	1.8 (0.3)	0.4	0.75
With horseradish	Water, red beetroot*, horseradish* 5%, garlic*, onion*, bay leaf*, allspice*, salt	12	<0.1 (<0.1)	1.8 (0.3)	0.4	0.75
With garlic	Water, red beetroot*, garlic*10%, bay leaf*, allspice*, kłodawska salt	12	<0.1 (<0.1)	1.8 (0.3)	0.4	0.75
With acerola	Water, red beetroot*, lemon*, acerola (1%), bay leaf*, allspice*, salt	12	<0.1 (<0.1)	1.8 (0.3)	0.4	0.75
Iodized	Red beetroot*, water, garlic*, allspice*, bay leaf*, salt, sodium iodide, potassium iodide	12	0 (0)	1.8 (0.3)	0.4	0.75
Without salt	Water, red beetroot*41%, garlic*, bay leaf*, allspice	12	<0.1 (<0.1)	1.8 (0.3)	0.4	0

* From organic farming.

**Table 2:** Antioxidant potential of the fermented beetroot juices tested using the FRAP method.

FRAP (µM Fe (II)/L)							
Type of Fermented Beetroot Juice	Minimum	25th Percentile	Median	75th Percentile	Maximum	K–W *p* Value	Different From *
(a) Without salt	5553.400	5634.350	5663.40	5690.500	5761.800	<0.000001	b–f
(b) Iodized	5464.100	5524.325	5538.60	5588.600	5670.600		a, c, f
(c) Traditional	5253.400	5280.900	5334.60	5371.225	5391.200		a, b, d–f
(d) With acerola	5433.900	5528.100	5623.20	5639.475	5677.400		a, c, e, f
(e) With horseradish	5406.000	5474.075	5511.00	5554.525	5625.800		a, c, d, f
(f) With garlic	5341.600	5358.550	5420.80	5471.250	5521.700		a–e

K–W—Kruskall–Wallis; *—different letters in the same column mean that the results differ significantly at *p* < 0.05. in the post-hoc test.

**Table 3:** Antioxidant potential of the fermented beetroot juices tested using the ABTS method.

ABTS (%)							
Type of Fermented Beetroot Juice	Minimum	25th Percentile	Median	75th Percentile	Maximum	K–W *p* Value	Different From *
(a) Without salt	96.320	96.526	96.613	96.672	96.708	<0.000001	b–f
(b) Iodized	94.009	94.105	94.236	94.380	94.418		a, c–f
(c) Traditional	62.089	65.479	69.943	70.631	71.353		a, b, d–f
(d) With acerola	48.171	49.157	54.369	61.475	61.837		a–c, e–f
(e) With horseradish	37.936	38.931	44.146	45.938	46.746		a–d, f
(f) With garlic	79.991	80.065	80.118	80.656	80.985		a–e

K–W—Kruskall–Wallis; *—different letters in the same column mean that the results differ significantly at *p* < 0.05 in the post-hoc test.

**Table 4:** Antioxidant potential of the fermented beetroot juices tested using the DPPH method.

DPPH (%)							
Type of Fermented Beetroot Juice	Minimum	25th Percentile	Median	75th Percentile	Maximum	K–W *p* Value	Different From *
(a) Without salt	54.756	55.112	65.143	65.285	65.442	<0.000001	b–f
(b) Iodized	73.452	73.470	73.489	74.041	74.408		a, c–f
(c) Traditional	76.929	77.174	78.149	79.026	79.066		a, b, d, e
(d) With acerola	73.897	73.939	73.959	74.824	74.967		a–c, e, f
(e) With horseradish	76.969	77.028	77.169	77.521	77.582		a–d
(f) With garlic	77.003	77.169	77.293	77.365	77.431		a, b, d

K–W—Kruskall–Wallis; *—different letters in the same column mean that the results differ significantly at *p* < 0.05 in the post-hoc test.

**Table 5:** Total polyphenol content (TPC) in the fermented beetroot juices.

TPC (mg GAE/L)							
Type of Fermented Beetroot Juice	Minimum	25th Percentile	Median	75th Percentile	Maximum	K–W *p* Value	Different From *
(a) Without salt	750.580	750.655	760.020	787.195	790.450	<0.000001	b–f
(b) Iodized	110.050	110.085	110.170	110.327	110.390		a, c–f
(c) Traditional	108.740	108.817	109.470	109.653	109.700		a, b, d
(d) With acerola	111.400	111.420	112.680	112.795	112.870		a–c, e, f
(e) With horseradish	108.770	108.827	108.930	110.280	110.350		a, b, d, f
(f) With garlic	107.770	107.797	107.840	109.888	109.960		a, b, d, e

K–W—Kruskall–Wallis; *—different letters in the same column mean that the results differ significantly at *p* < 0.05 in the post-hoc test.

**Table 6:** Total flavonoid content (TFC) in the fermented beetroot juices.

TFC (mg RE/L)							
Type of Fermented Beetroot Juice	Minimum	25th Percentile	Median	75th Percentile	Maximum	K–W *p* Value	Different From *
(a) Without salt	205.330	206.337	221.280	222.180	223.420	<0.000001	b–f
(b) Iodized	83.922	87.335	89.931	96.246	97.320		a, c–e
(c) Traditional	101.650	106.068	108.090	109.278	110.290		a, b, f
(d) With acerola	102.660	105.197	112.020	114.473	115.340		a, b, e, f
(e) With horseradish	98.296	99.371	104.020	108.830	109.090		a, b, d, f
(f) With garlic	87.532	90.213	94.552	107.982	108.760		a, c–e

K–W—Kruskall–Wallis; *—different letters in the same column mean that the results differ significantly at *p* < 0.05 in the post-hoc test.

**Table 7:** Total vitamin C content in the fermented beetroot juices.

Vitamin C (mg/100 mL)							
Type of Fermented Beetroot Juice	Minimum	25th Percentile	Median	75th Percentile	Maximum	K–W *p* Value	Different From *
(a) Without salt	29.870	29.903	29.927	29.933	29.966	<0.000001	b–f
(b) Iodized	45.627	45.818	51.859	55.879	55.905		a, c–f
(c) Traditional	39.316	39.370	41.669	43.253	43.371		a, b, d, f
(d) With acerola	38.470	38.515	46.358	64.905	64.962		a–c
(e) With horseradish	38.982	39.076	44.678	46.019	46.057		a, b
(f) With garlic	38.530	38.802	47.885	47.932	48.018		a–c

K–W—Kruskall–Wallis; *—different letters in the same column mean that the results differ significantly at *p* < 0.05 in the post-hoc test.

**Table 8:** The pH of the fermented beetroot juices.

pH						
Type of Fermented Beetroot Juice	Minimum	25th Percentile	Median	75th Percentile	Maximum	K–W *p* Value
(a) Without salt	3.90	3.90	3.91	3.96	3.98	0.055899
(b) Iodized	3.39	3.39	3.40	3.41	3.41	
(c) Traditional	3.50	3.50	3.50	362	3.66	
(d) With acerola	3.42	3.44	3.49	3.49	3.49	
(e) With horseradish	3.53	3.57	3.67	3.67	3.67	
(f) With garlic	3.48	3.49	3.52	5.02	5.52	

**Table 9:** Sensory evaluation of the fermented beetroot juices.

Sensory Evaluation						
Type of Fermented Beetroot Juice	Aroma	Sweetness	Acidity	Colour	Overall Acceptability	K–W *p* Value
(a) Without salt	7.60	7.40	7.40	8.80	8.20	0.41588
(b) Iodized	7.80	7.40	7.20	8.40	7.60	
(c) Traditional	7.83	6.67	7.80	8.33	7.83	
(d) With acerola	6.80	6.00	5.80	8.80	7.00	
(e) With horseradish	8.25	7.50	8.00	9.00	8.00	
(f) With garlic	7.60	7.20	7.40	8.80	8.00	

## Data Availability

The data presented in this study are available on request from the corresponding author. The data are not publicly available due to the data design into other ongoing research should be protected before formal publication.
